# Regarding: ‘prediction of postoperative nausea and vomiting in patients undergoing sedated gastrointestinal endoscopy based on machine learning’

**DOI:** 10.1080/07853890.2025.2611193

**Published:** 2026-01-05

**Authors:** Chenhui Lv, Qijia Xuan

**Affiliations:** International Institutes of Medicine, Fourth Affiliated Hospital, School of Medicine, Zhejiang University, Yiwu, China

We read with considerable interest the multicentre study by Yao and colleagues that applied machine-learning approaches to predict postoperative nausea and vomiting (PONV) after sedated gastrointestinal endoscopy [1]. By developing and validating 11 models, selecting an LDA model with favourable generalisability, leveraging SHAP to enhance interpretability, and deploying an online prediction tool, the authors provide a potentially useful strategy for precision risk assessment and more individualised perioperative management.

First, the diversity of the study population and the selection/definition of predictors could be further strengthened. The external validation cohort was derived exclusively from hospitals within the same geographic region in China, which may limit transportability across populations with different ethnic backgrounds, healthcare resources, and perioperative practice patterns [2]. In addition, several clinically important predictors were not captured, including genetic susceptibility (e.g. CYP2D6 polymorphisms associated with PONV vulnerability), intraoperative antiemetic prophylaxis, and granular anaesthetic details (specific agents and doses). This is particularly relevant in sedated endoscopy, where PONV risk is strongly influenced by sedation strategy and medication choices (e.g. propofol vs remimazolam selection and dosing, concomitant opioid use and dose, sedation depth and bolus patterns), as well as by prophylactic and rescue antiemetic use—factors that may act as strong confounders and materially affect model portability across centres [3,4]. We suggest that the authors, at minimum, provide a structured description of sedation protocols and antiemetic prevention/rescue pathways (indications, drug classes/doses, and timing). Where data permit, incorporating key medication variables into modelling or performing sensitivity analyses (with vs without antiemetic-related variables, comparing discrimination and calibration) would help clarify whether the model estimates ‘intrinsic/natural’ risk or the residual risk under a specific prophylaxis strategy, thereby improving interpretability and clinical usability.

Second, deeper and more clinically anchored model comparisons would enhance the manuscript’s impact. Because the evaluation did not include head-to-head benchmarking against widely used conventional tools (e.g. the simplified Apfel score), it remains difficult to quantify incremental value in predictive performance, operational simplicity, and personalised adaptability—areas where traditional scores often have advantages in uptake and workflow feasibility [5]. We recommend direct comparisons using consistent metrics (e.g. AUC, calibration, and—where feasible—time burden and user acceptability) to delineate the model’s competitive strengths and its appropriate boundary of use. Moreover, additional discussion is warranted regarding why LDA emerged as the ‘optimal’ model given the potentially complex and non-linear relationships in clinical data, alongside clearer assessment of overfitting risk and benchmarking against non-linear/deep-learning approaches in the same task.

In summary, this work introduces an interpretable, web-deployable ML-based model for PONV risk prediction after sedated gastrointestinal endoscopy, with meaningful innovation and translational potential. Further improvements in population diversity, inclusion and clarification of key perioperative medication variables, and more rigorous comparisons with established clinical tools would likely strengthen generalisability, scientific robustness, and real-world applicability.

## Data Availability

Data availability is not applicable to this article as no new data were created or analyzed in this study.

## References

[1] Yao Y, Li Y, Xing F, et al. Prediction of postoperative nausea and vomiting in patients undergoing sedated gastrointestinal endoscopy based on machine learning. Ann Med. 2025;57(1):2570792. doi:10.1080/07853890.2025.2570792. 41355079 PMC12687890

[2] Goldson KV, Brennan E, Burton BN, et al. Does management of postoperative nausea and vomiting differ by patient demographics? An evaluation of perioperative anesthetic management-an observational study. Anesthesiology. 2025;142(4):704–715. doi:10.1097/ALN.0000000000005367. 39786950

[3] Wang D, Sun Y, Zhu YJ, et al. Comparison of opioid-free and opioid-inclusive propofol anaesthesia for thyroid and parathyroid surgery: a randomised controlled trial. Anaesthesia. 2024;79(10):1072–1080. doi:10.1111/anae.16382. 39037325

[4] White RS, Andreae MH, Lui B, et al. Antiemetic administration and its association with race: a retrospective cohort study. Anesthesiology. 2023;138(6):587–601. doi:10.1097/ALN.0000000000004549. 37158649

[5] Violante T, Ferrari D, Novelli M, et al. Super learner enhances postoperative complication prediction in colorectal surgery. Ann Surg. 2025. doi:10.1097/SLA.0000000000006847. 40698768

